# The Florence Nightingale Memorial

**Published:** 1916-02-12

**Authors:** 


### The Hospital

The Workers' Newspaper of Administrative Medicine and Institutional Life, Administration, National Insurance and Health.

No. 1548, Vol. LIX. SATURDAY, FEBRUARY 12, 1916. PRICE ONE PENNY.

### THE FLORENCE NIGHTINGALE MEMORIAL.

Delays are dangerous, but they may sometimes prevent indifferent work, and end in results greater and better than was thought possible at the outset. Such, in a sentence, has been the outcome of the work of the committee to secure a fitting memorial to Florence Nightingale. How true this is may be realised by anyone who will turn to the leading article on this memorial in The Hospital of October 21, 1911. This leader was written a year after the memorial was started, when less than half the money originally asked for was forthcoming. We ventured then to make an urgent appeal to hospital workers, on the ground that the inspiration of Florence Nightingale's work had given to all of them a living force which had influenced the lives of hundreds of thousands among us to their infinite uplifting and benefit. In the result the £5,000 originally asked for was raised to £8,000, a sum which has enabled the statue to be erected in Pall Mall, and has provided some much-needed pensions for nurses under special and suitable conditions. Further, the wish expressed by many subscribers, and voiced by us, that efforts should be made to place a marble plaque of sculpture in bas-relief in St. Paul's Cathedral, and if possible in Westminster Abbey, will attain fulfilment on Monday, 14th instant, when Her Majesty the Queen is to unveil in St. Paul's Cathedral the most beautiful work which Mr. A. G. Walker, the able sculptor, has produced. It has not yet been found possible to place a similar plaque in Westminster Abbey, owing to the requirement, as an initial step, that £200 shall be paid to the Abbey Restoration Fund. We insisted that the contributions to the general memorial should be raised by the small offerings of the workers who have been inspired by Florence Nightingale's spirit. This has been done, and now there is an opportunity for one or two rich people who realise the importance of Florence Nightingale's work for the nation to come forward and overcome the pecuniary difficulty which has so far excluded from Westminster Abbey a Florence Nightingale plaque.

Great disappointment and some criticism arose when, owing to the war and the political and other difficulties attending it, the statue to Florence Nightingale erected in Pall Mall was not publicly unveiled with due ceremony. It would fulfil no useful purpose to dwell upon the difficulties in question, or to examine them in detail. The fact remains that, after the fullest consideration, it was found impossible to follow this plan, to the great regret of the Memorial Committee, of the Government, and of all the many friends and disciples of Florence Nightingale. Let it suffice here to record that any such attempt at that time might have been attended by results so far-reaching and undesirable as to defeat the very object, upon the fulfilment of which alone must rest the desirability of a public ceremony on such an occasion. Anyone who challenges this conclusion will fail in judgment and display a lamentable want of knowledge and apprehension.

In these circumstances Her Majesty the Queen's consent to unveil the plaque at St. Paul's Cathedral on the 14th instant is most welcome. The occasion will be one of Imperial importance and the ceremonial and after-service will fittingly commemorate Florence Nightingale's work and the inspiration it was and will give to all workers among the sick. The Archbishop of Canterbury, who is ever foremost in emphasising the importance he attaches to personal service in the present day, will give a short address. The Chairman of the Memorial Committee, the Earl of Pembroke, is specially coming from the trenches to be present at the ceremony, a fact worthy of the son of a father who was the first chairman of this Memorial Committee and did a really splendid work in this connection, which had much to do with the notable success achieved. Lord Crewe will also attend, and either Lord Crewe or Lord Pembroke will present the sculptor, Mr. A. G. Walker, to the Queen. There will not be room in the Crypt of St. Paul's Cathedral to accommodate more than the members of the Memorial Committee, the Matrons-in-Chief of the Navy and Army, and the matrons of several important hospitals, who will fittingly represent the whole nursing profession. Immediately after the ceremony in the Crypt a special service open to the public will be held in the Cathedral, where some 1,500 seats will be reserved for matrons, sisters, and nurses in uniform. Among the contributors to the memorial have been many of His Majesty's ships and regiments, and it is hoped that both Services will be adequately represented by men in uniform, wounded or otherwise. There is every prospect that the occasion may become notable in history, and prove in every way worthy of the important work and results achieved by Florence Nightingale during a long and useful life.

Had Florence Nightingale lived she would, we are confident, from personal knowledge, have welcomed Mr. Arthur Stanley's proposal to unite all parties in the nursing world by establishing a College of Nursing. Such a College, organised on the careful and acceptable lines which Mr. Stanley is labouring so ably and conscientiously to secure, with the consent and co-operation of the trusted representatives and leaders of the nursing profession in this country, promises to eventuate in an organisation which will provide for nurses of all ranks the recognition and advantages they have so long desired. These include the government of the profession by the profession, a uniformity of curriculum, a statutory examination, and either registration or a system which can better guarantee and secure all that is claimed for the latter. We receive daily encouraging proofs of the widespread interest Mr. Stanley's scheme has excited, of the confidence his name has brought to the Nursing College scheme, and of the determination of those most concerned to co-operate with him in securing for it the maximum of success.

